# Prophylactic ultra-low dose rituximab to maintain remission in relapsing adult minimal change disease

**DOI:** 10.1093/ckj/sfad270

**Published:** 2023-10-19

**Authors:** Wing Yin Leung, Henry H L Wu, Alexander Woywodt, Arvind Ponnusamy

**Affiliations:** Department of Renal Medicine, Lancashire Teaching Hospitals NHS Foundation Trust, Preston, Lancashire, UK; Renal Research, Kolling Institute of Medical Research, Royal North Shore Hospital & The University of Sydney, Sydney, NSW, Australia; Department of Renal Medicine, Lancashire Teaching Hospitals NHS Foundation Trust, Preston, Lancashire, UK; Faculty of Biology, Medicine & Health, The University of Manchester, Manchester, UK; Department of Renal Medicine, Lancashire Teaching Hospitals NHS Foundation Trust, Preston, Lancashire, UK; Faculty of Biology, Medicine & Health, The University of Manchester, Manchester, UK

To the Editor,

Rituximab has been used for the management of relapsing adult minimal change disease (MCD) but the optimal regime and dose remain unclear. Recent reports described favourable outcomes with ultra-low dose rituximab (i.e. 200 or 250 mg/dose). We present two additional cases where this novel, cost-effective and convenient strategy was used successfully.

**Figure 1:**
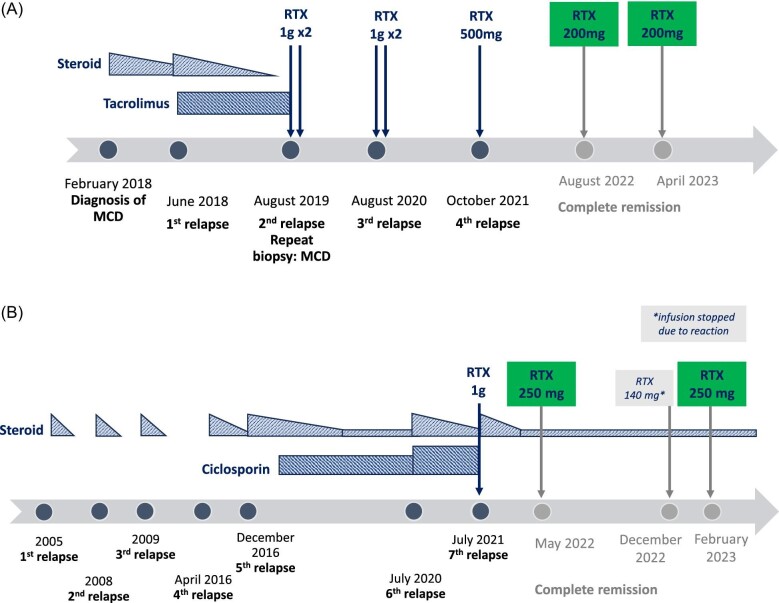
Timeline illustrating the clinical course of minimal change disease and immunosuppressive treatment received (A: first case; B: second case). MCD: minimal change disease; RTX: rituximab.

A 76-year-old woman presented in February 2018 with nephrotic syndrome (uPCR 1177 mg/mmol, serum albumin 13 g/L) and was diagnosed with MCD following kidney biopsy (Fig. 1A). She was commenced on prednisolone 60 mg daily which was gradually weaned down. The patient had her first relapse in June 2018 while taking prednisolone 5 mg daily. A course of increased dose prednisolone was given and tacrolimus was added. The second relapse occurred in August 2019, two months following cessation of steroid treatment but while on tacrolimus. The patient then commenced on two doses of rituximab 1 g six weeks apart and tacrolimus was discontinued. A third relapse occurred in August 2020, and two doses of rituximab 1 g were administered, which achieved remission. Her fourth relapse occurred in October 2021, and she received another 500 mg of rituximab as single dose. As a plan to prevent further relapses, the patient was prescribed a further two doses of rituximab 200 mg as guided by her CD19 B-cell repopulation. Her recent rituximab dose was in April 2023 and CD19 count remains depleted at 1 cell/µL. The patient did not have further relapses to date and eGFR is stable at 60 ml/min/1.73 m^2^.

The second case was a 61-year-old woman with relapsing MCD since 2005 (Fig. 1B). She achieved complete remission with steroid treatment for her first five relapses between 2005 and 2016. Ciclosporin was commenced in March 2017, keeping her in remission until July 2020 when she experienced her sixth relapse. She was then treated with increased prednisolone and ciclosporin doses. The patient's seventh relapse in July 2021 resulted in the decision to convert her treatment from ciclosporin to rituximab 1 g single dose alongside another course of weaning prednisolone treatment. To prevent further relapses, the patient was scheduled to receive rituximab 250 mg at six-monthly intervals and to remain on prednisolone 5 mg daily. This regime was continued. Rituximab was last administered at the end of February 2023 and her CD19 count remains depleted. The patient did not have further relapses and remains in remission with a stable eGFR >90 ml/min/1.73 m^2^.

To our knowledge, there are only two studies to date that have reported the use of ultra-low dose rituximab in adult MCD. Zhang *et al.* [1]. studied the efficacy of ultra-low dose rituximab in treatment and prevention of frequently relapsing MCD, whileFujimoto *et al.* [2] evaluated ultra-low dose rituximab in maintaining remission. Both studies suggested that rituximab dosing at 200 mg/dose may reduce MCD relapse rates and fewer adverse events with concurrent steroid administration [1, 2]. In the study by Fuijmoto *et al.* [2], it is an interesting observation that CD19 count was not depleted in vast majority of cases (90.5%). This postulates a pharmacodynamic mechanism that may be independent of B-cell repopulation.

It is important to note differences between membranous nephropathy and MCD as proteinuria is highly selective in the latter, leading to fewer urinary losses when rituximab is given. A preliminary study by Guan *et al.* [3], which investigated rituximab use without concurrent steroid administration in nine patients with MCD suggested that more severe nephrotic patients with MCD may require higher doses of rituximab and that dosing should be more individualized. Our two cases reported here were not severely nephrotic and we speculate that this is why the ultra-low dose regime worked so well for them. We suggest that further studies should confirm the utility of ultra-low dose and individualized rituximab dosing in relapsing MCD.
